# Treatment preference for once-weekly versus once-daily DPP-4 inhibitors in patients with type 2 diabetes mellitus: a systematic review and meta-analysis of randomized controlled trials

**DOI:** 10.1080/07853890.2025.2603036

**Published:** 2025-12-26

**Authors:** Xingxing Xie, Yu Chen, Pei Wang, Ming Hu

**Affiliations:** ^a^ West China School of Pharmacy, Sichuan University, Chengdu, Sichuan Province, China; ^b^ Department of Pharmacy, Ya’an People’s Hospital, Ya’an, Sichuan Province, China

**Keywords:** Type 2 diabetes mellitus, treatment preference, once-weekly DPP-4 inhibitors, once-daily DPP-4 inhibitors, randomized controlled trials, systematic review

## Abstract

**Background/Objective:**

Although once-weekly and once-daily DPP-4 inhibitors have gained widespread market recognition, patient preference differences remain a key focus. This meta-analysis compares treatment preferences for once-weekly versus once-daily DPP-4 inhibitors in T2DM, offering evidence to guide clinical decisions and healthcare policies.

**Methods:**

PubMed, OVID, EBSCO, Web of Science, CNKI, Wanfang, and clinical trial registries were searched up to June 30, 2025. After screening literature against predefined criteria, a systematic review was conducted to compare the effects of once-weekly and once-daily DPP-4 inhibitors on the treatment preferences of patients with T2DM.

**Results:**

8 RCTs with 1,575 participants were analyzed. No significant difference in medication adherence and DTSQ total score between the once-weekly and once-daily groups (*p* > 0.05). HbA1c percentage (MD = -0.21, 95% CI [-0.42, -0.01], *p* < 0.05) decreased significantly with once-weekly dosing, while GA and FPG showed no change (*p* > 0.05), this suggests greater improvement in HbA1c percentage levels following a switch to once-weekly DPP-4 inhibitors. Once-weekly DPP-4 inhibitors showed higher musculoskeletal/connective tissue disorder risk (RR = 2.63; 95% CI [1.18, 5.83]), but no significant differences in other adverse events (*p* > 0.05). No significant differences in treatment burden between both groups (*p* > 0.05).

**Conclusion:**

No statistically significant association between treatment preferences for once-weekly versus once-daily DPP-4 inhibitors among T2DM patients and medication adherence, treatment satisfaction, glycemic level changes, safety, or treatment burden for these two dosing regimens. Further research is needed to elucidate the influence of physician prescribing behavior on these preferences.

## Introduction

Type 2 diabetes mellitus (T2DM) is the most prevalent diabetes subtype worldwide [1], and it constitutes a major 21st-century public health challenge [2]. Between 1990 and 2021, the global incidence of T2DM increased from 56.02 to 123.86 cases per 100,000 population—approximately a 1.5-fold rise [3]. By 2045, global prevalence is projected to reach 12.2% (783.2 million adults) [4], predominantly driven by increases in developing countries, with prevalence peaking at 13.1% [4,5]. T2DM pathogenesis is complex, encompassing insulin resistance [6], β-cell dysfunction, chronic inflammation, endoplasmic reticulum stress, and oxidative stress [7,8].

Current T2DM prevention and treatment strategies emphasize lifestyle modification, pharmacotherapy, and surgical treatment [9]. Among these, comprehensive management is essential to improve patient quality of life and mitigate complications [10,11]. When lifestyle modifications fail to achieve glycemic control, pharmacotherapy becomes necessary, encompassing metformin, sulfonylureas, glinides, α-glucosidase inhibitors, thiazolidinediones, dipeptidyl peptidase-4 (DPP-4) inhibitors, sodium-glucose cotransporter-2 inhibitors, and glucagon-like peptide-1 receptor agonists. Although the traditional oral antidiabetic agents such as metformin and sulfonylureas are used less frequently in high-risk patients (such as those with heart failure or chronic kidney disease), their prescription remains relatively high for newly diagnosed patients with T2DM [12].

Over the past 20 years, despite significant advances in the medical treatment of patients with diabetes, the risk of hypoglycemia, weight gain, and high incidence of cardiovascular disease and related complications remain the leading causes of mortality or disability in patients with T2DM [13]. Approximately 40% of patients with T2DM will develop diabetic nephropathy, and 70% of those aged ≥ 65 die from cardiovascular diseases [14]. The therapeutic limitations of traditional oral antidiabetic drugs are affecting patients’ health expectations. Therefore, T2DM treatment must simultaneously consider cardiovascular protection and prevention of complications. DPP-4 inhibitors—a novel class of oral hypoglycemic agents—increase endogenous active GLP-1 levels two- to three-fold by inhibiting DPP-4. This consequently enhances β and α cell glucose sensitivity[15]. These agents are characterized by rapid oral absorption, long half-life, and long-lasting efficacy [15], and also confer cardiovascular and renal protection [16–18]. Since the approval of the first DPP-4 inhibitor, sitagliptin, in October 2006, more than 10 innovative agents have been introduced in China, the United States, and Japan, and have all received widespread market recognition. Industry projections estimate the global DPP-4 inhibitor market will surpass 10 billion US dollars by 2030.

Currently, most DPP-4 inhibitors are once-daily dosage forms. However, with the approval of the first once-weekly DPP-4 inhibitor, trelagliptin, new treatment options have been provided for patients with T2DM. Meanwhile, other novel once-weekly DPP-4 inhibitors, such as omarigliptin, SYR-472, and HSK7653, also demonstrate favorable efficacy and safety in clinical trials [19–21]. This represents that competing products in the same category are developing rapidly and accelerating their time to market in order to meet market demand and enrich treatment options for patients. A study [30] based on treatment selection rate, changes in DTSQ total score and HbA1c level relative to baseline, treatment adherence, and safety conducted a pooled analysis of treatment preferences for once-weekly and once-daily DPP-4 inhibitor therapy in patients with T2DM. The results showed that patients had a significantly higher preference for once-daily treatment than once-weekly treatment, although patient satisfaction and HbA1c levels were similar across treatments. As we all know, in the management of T2DM, patients’ treatment preferences are crucial for long-term medication adherence and efficacy, and may even serve as an important factor in improving prognosis. Therefore, the aforementioned research provides us with a good idea. We believe it is necessary to conduct a systematic review study from the perspective of individual differences in medication selection, in order to explore patients’ treatment preferences for the once-weekly and once-daily dosing regimens of currently marketed DPP-4 inhibitors. By including randomized controlled trials, adopting strictly designed evidence-based medical methods, and comparing patient treatment preferences between once-weekly and once-daily DPP-4 inhibitors based on indicators such as patient adherence and treatment satisfaction, this study aims to provide more reliable evidence-based medical evidence for clinical drug treatment decision-making and medical insurance policy formulation.

## Materials and methods

### Protocol and registration

This systematic review was conducted in accordance with the Preferred Reporting Items for Systematic Reviews and Meta-Analyses (PRISMA 2020) guidelines [22]. This systematic review has been based on randomized controlled trials and prospectively registered in the PROSPERO database (registration number: CRD420251107529).

### Inclusion criteria

#### Type of study

Cohort studies offer a more comprehensive assessment of patient-centered outcomes—including treatment adherence, satisfaction, and health-related quality of life improvements—than randomized controlled trials (RCTs). However, these two study designs differ substantially in terms of bias profiles, causal inference capacity, and confounding adjustment strategies. Consequently, only data derived from RCTs were included in the concurrent analysis. Only studies published in English or Chinese were included.

#### Study population

Eligible individuals were adults (≥18 years old) clinically diagnosed with T2DM [23] and had used traditional antidiabetic medication such as metformin and sulfonylureas during the treatment process or were currently undergoing treatment with DPP-4 inhibitors, administered once-weekly or once-daily, either as monotherapy or in combination. All patients included were part of the full analysis set, and they were primarily drawn from English- and Chinese-speaking countries.

#### Intervention measure

The intervention group was administered a DPP-4 inhibitor once weekly, while the control group was administered daily dosing of the same medication (once daily).

#### Outcome indicators

During the follow-up period, the primary outcomes were the medication adherence (By using an electronic medication record card) [24], treatment satisfaction (Diabetes Treatment Satisfaction Questionnaire, DTSQ) [24], and changes from baseline levels in glycated hemoglobin (HbA1c percentage), glycoalbumin (GA), and fasting plasma glucose (FPG) levels among patients switching to DPP-4 inhibitor once-weekly and those continuing to daily dosing. The secondary outcomes comprised incidence of adverse events—including drug-related treatment-emergent adverse events (TEAEs), serious adverse events (SAEs) leading to discontinuation, infections, infestations, gastrointestinal disorders, musculoskeletal and connective tissue disorders, and viral upper respiratory tract infections. Another indicator is the treatment burden of patients, and the treatment burden indicator statistics uniformly adopt the Diabetes Treatment Burden Questionnaire (DTBQ) [35].

#### Exclusion criteria

The exclusion criteria were as follows: 1)nonrandomized intervention studies, 2) lack of valid data, 3) inconsistent measurement statistics of relevant indicators and inability to convert, 4) reviews and clinical overviews, 5) data not aligned with research objectives, and 6) literature with excessive data heterogeneity.

#### Search strategy

PubMed (https://pubmed.ncbi.nlm.nih.gov/), OVID (https://ovidsp.ovid.com/), EBSCO (https://www.ebsco.com/), China National Knowledge Infrastructure (CNKI) (https://www.cnki.net/), and Wanfang Medical Network (https://www.wanfangdata.com.cn/), as well as relevant clinical trial registries, were searched for RCTs of once-weekly versus once-daily DPP-4 inhibitors in patients with T2DM. The search encompassed all records through June 30, 2025, including both databases and clinical trial registries. Grey literature trial registries supplemented published results. Search terms were formulated in Chinese and English, combining homonyms, subject words, and free-texts, with default database expansion retrieval performed, supplemented by manual retrieval (literature tracking). The following terms were searched: type 2 diabetes mellitus, T2DM, once-weekly DPP-4 inhibitor, once-daily DPP-4 inhibitor, sitagliptin, saxagliptin, linagliptin, alogliptin, vildagliptin, trelagliptin, omarigliptin, active-controlled, parallel-group, randomized clinical trial (or RCT), prospective and retrospective randomed-control studies, medication adherence, treatment satisfaction, treatment burden, HbA1c percentage, and blood glucose levels. In the PubMed database, the following terms were combined using PICOS-based Boolean operators:

(“diabetes mellitus, type 2” [MeSH] OR “T2DM” OR “type 2 diabet*”) AND (“once-weekly DPP-4 inhibitor” [MeSH] OR “trelagliptin” OR “omarigliptin”) AND (“once-daily DPP-4 inhibitor” [MeSH] OR “daily DPP-4” [MeSH] OR “sitagliptin” [MeSH]) AND (“medication adherence” [MeSH] OR “treatment satisfaction” [MeSH] OR “treatment burden” OR “blood glucose levels” [MeSH]) AND (“randomized controlled trial”[Publication Type] OR “random*” OR “randomed-control studies” [MeSH]).

#### Literature screening and data extraction

To minimize selection bias, two investigators (XX and WP) independently screened the literature against eligibility criteria and extracted relevant data; any discrepancies were analyzed and resolved by the third investigator (YC). When multiple reports presented identical data, the highest-quality source was selected [25].

#### Literature quality evaluation

The risk of bias in RCT was assessed using the Cochrane Manual for Systematic Review of Interventions version 5.1.0 [26]. A three-level risk classification (low bias risk, unclear risk, and high bias risk) was employed to evaluate the following domains: the method of random sequence generation, the description degree of blinding, assignment concealment (including any unblinded procedure), outcome data integrity (such as exit/loss rate), selective reporting bias risk, and other potential sources of bias. A low bias risk indicates high reliability of the literature data.

### Statistical analysis

#### Analysis method

Meta-analyses for each effect indicator were performed using RevMan 5.4 software, as recommended by the Cochrane Collaboration network (http://www.cochrane.org). Statistical effect sizes included odds ratio (OR), risk ratio (RR) for categorical data, and standardized mean difference (SMD) or mean difference (MD) for continuous data, each with corresponding 95% confidence intervals (CI). A Q-test was used to evaluate heterogeneity across studies. Studies with *p* ≥ 0.05 or *I*^2^ ≤ 50% were considered homogenous, and all studies could be combined for meta-analysis using the fixed effects model. Conversely, the random effects model (REM) was used for meta-analysis. Publication bias was evaluated using a funnel plot for outcomes included in more than five studies. Sensitivity analyses were used to verify the results of systematic evaluation, supplemented by descriptive analyses, where appropriate.

## Results

### Literature search results

**Figure 1.**
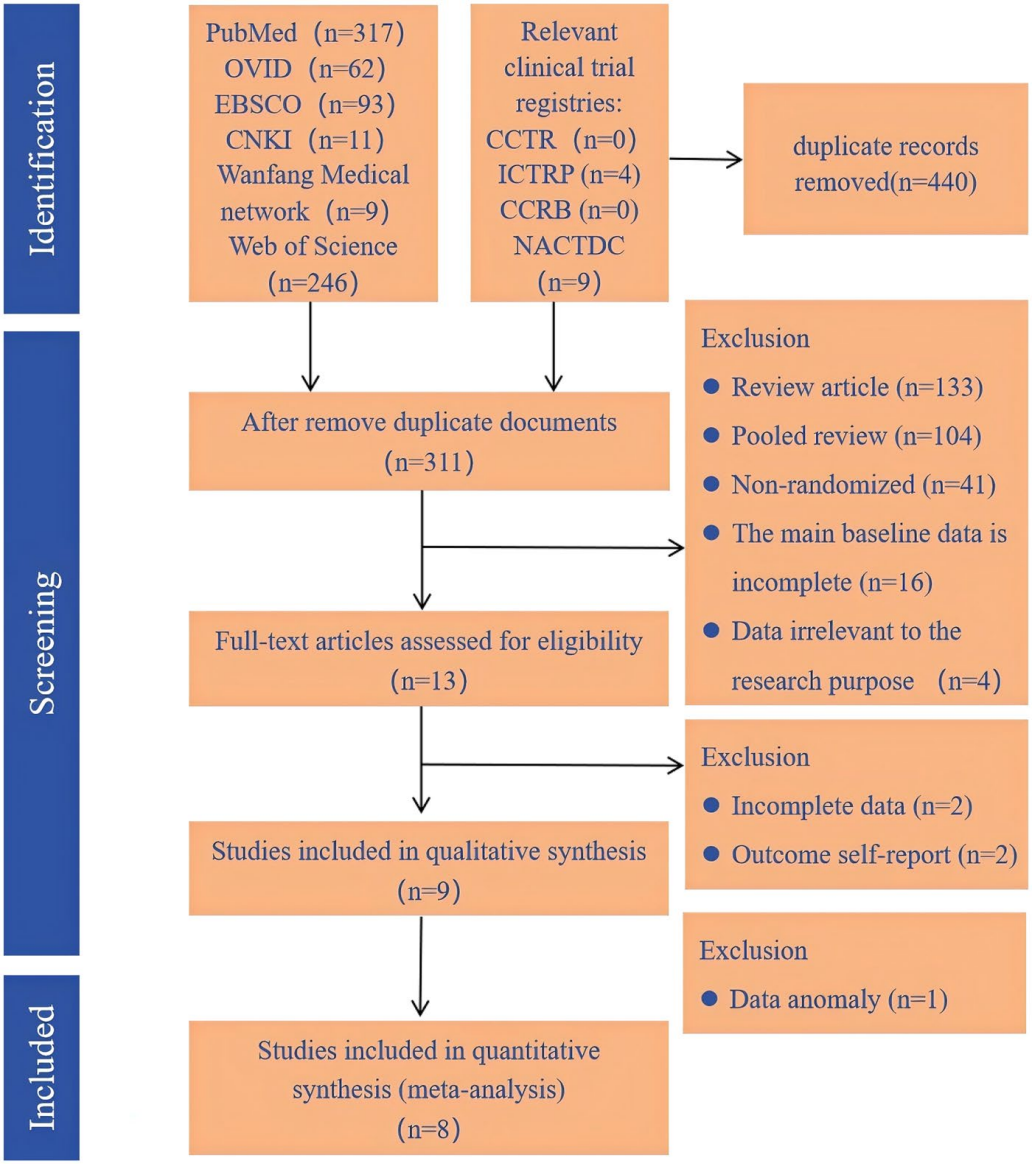
The PRISMA fowchart for the selection and screening process.

A total of 751 studies were initially retrieved using the predefined search strategy. Following deduplication, 440 studies were excluded, leaving 311 for further screening. During the eligibility assessment, 298 studies were eliminated for multiple reasons: 278 were non-RCTs, reviews, or pooled analyses; 16 lacked complete baseline data; and 4 were irrelevant to the research objective. Full-text evaluation was conducted on the remaining 13 studies, of which 2 were excluded due to incomplete data, 2 for self-reported outcomes, and 1 for data anomalies. Ultimately, eight English-language studies were included in the final analysis [27–34]. (Figure 1).

### Literature features

#### Basic information

According to the inclusion and exclusion criteria, 8 RCTs [27–34] were selected. The basic data of the literature were complete, and the baseline characteristics—age, sex, BMI, intervention measures, and glycemic levels—were balanced, with no statistically significant difference (*p* > 0.05), confirming comparability (Table 1).

**Table 1.** Basic features of the included literature (mean ± SD).

Author and year of publication (Ref.)	Study design	Controlled intervention	Patient (n)	Age (years)	Male (n,%)	Body weight (kg)	BMI (kg/m^2^)	HbA1c (%)	GA (%)	FPG (mmol/L)	Duration of diabetes mellitus (years)	Observation time (week)	DTSQ total score (excluding Q2 and Q3)
Ishii (27)	RCT	Trelagliptin 100 mg	110	61.5 (8.96)	86 (78.2)	—	24.72 (3.224)	7.40 (0.819)	—	8.33 (1.55)	6.45 (4.972)	12	22.31 (5.444)
2019		Daily DPP-4	108	58.4 (10.01)	82 (75.9)	—	25.53 (4.349)	7.34 (0.620)	—	8.40 (1.70)	6.39 (5.286)		21.56 (5.679)
Ohara (28)	RCT	Omarigliptin 25 mg	18	66.8 (6.6)	13 (72.2)	61.6 (9.8)	23.3 (3.2)	7.2 (0.4)	7.9 (1.2)	7.9 (1.2)	11.9 (7.6)	24	26.4 (5.5)
2021		Daily DPP-4	18	69.0 (9.2)	12 (66.7)	68.0 (12.5)	25.6 (3.5)	7.2 (0.4)	7.6 (0.94)	7.6 (0.94)	14.5 (6.5)		25.3 (7.2)
Meguro (29)	RCT	Trelagliptin 100 mg	30	59.7 (8.73)	28 (93.3)	—	24.19 (3.31)	7.41 (0.84)	—	—	—	16	23.0 (5.48)
2019		Alogliptin 25 mg	30	60.6 (8.86)	28 (93.3)	—	24.61 (3.22)	7.34 (0.63)	—	—	—		23.7 (5.86)
Tosaki (30)	RCT	Once-weekly DPP-4	29	63.9 (13.7)	16 (55.2)	62.1 (12.2)	24.2 (4.0)	6.98 (1.27)	20.3 (5.5)	—	—	12	21.9 (8.7)
2017		Daily DPP-4	37	52.0 (15.1)	26 (70.3)	69.6 (13.8)	25.6 (4.4)	9.31 (2.53)	26.7 (11.8)	—	—		24.1 (8.5)
Yoshizawa (31)	RCT	Omarigliptin 25 mg	14	67.7 (8.9)	12 (85.7)	—	—	6.2 (0.9)	18.8 (4.3)	—	16.0 (8.7)	24	—
2021		Linagliptin 20 mg	16	67.5 (9.0)	12 (75.0)	—	—	6.5 (1.0)	21.9 (3.8)	—	20.8 (11.3)		—
Inagaki (32)	RCT	Trelagliptin 100 mg	101	58.0 (9.63)	73 (72.3)	69.23 (14.27)	25.4 (4.42)	7.73 (0·85)	21.03 (3.45)	8.74 (1.68)	6.25 (5.94)	12	—
2015		Alogliptin 25 mg	92	60.0 (8.89)	69 (75.0)	67.37 (13.17)	24.7 (3.79)	7.87 (0.86)	22.07 (4.08)	9.22 (2.29)	7.07 (5.93)		—
Goldenberg (33)	RCT	Omarigliptin 25 mg	322	57.0 (10.0)	151 (46.9)	91.3 (20.1)	32.7 (6.1)	7.5 (0.8)	—	8.9 (2.0)	7.0 (4.5)	24	—
2017		Sitagliptin 50 mg	320	58.0 (10.0)	175 (54.7)	87.7 (16.9)	31.3 (5.1)	7.5 (0.7)	—	8.5 (1.8)	7.5 (5.6)		—
Gantz (34)	RCT	Omarigliptin 25 mg	166	60.0 (11.0)	104 (62.7)	67.0 (13.0)	25.2 (3.7)	7.9 (0.7)	—	9.0 (1.7)	—	24	—
2017		Sitagliptin 50 mg	164	60.0 (9.0)	115 (70.1)	69.0 (14.0)	25.4 (4.2)	8.0 (0.8)	—	8.8 (1.7)	—		—

RCT: Randomized controlled trial; ‘—’,unavailable; BMI: body mass index; HbA1: glycosylated hemoglobin A1c; GA: glycoalbumin; FPG: fasting blood glucose; DTSQ: diabetes treatment satisfaction questionnaire. The DTSQ scores were analyzed separately for treatment satisfaction and perceived hyperglycemia/hypoglycemia. Treatment satisfaction was estimated by scoring questions 1 (Satisfied), 4 (Convenient), 5 (Flexible), 6 (Understanding), 7 (Recommend) and 8 (Continue) of the DTSQ. Perceived hyperglycemia and hypoglycemia were expressed as the scores of questions 2 and 3, respectively.

#### Quality characteristics

The Cochrane Handbook for Systematic Reviews of Interventions version 5.1.0 tool was used to qualitatively assess each item of the 8 enrolled studies [27–34]. Results: All studies employed strict random-sequence generation and were rated as low risk of bias (L). However, four studies [27–29,31] were rated as high risk of bias (H) in the blinding of participants and personnel. Additionally, the research of Ishii [27] and Yoshizawa [31] also showed a high risk of bias (H) for ‘allocation concealment.’ No high risk was observed for selective reporting and other bias domains (Table 2) (details were shown in Supplementary Material 1).

**Table 2.** Quality evaluation of literature methodology.

Author and year of publication (Ref.)	Random sequence generation	Blinding of participants and personnel	Allocation concealment	Incomplete outcome data	Selective reporting	Other bias
Ishii 2019. (27)	L	H	H	L	U	L
Ohara 2021. (28)	L	H	U	L	U	L
Meguro 2019. (29)	L	H	U	L	L	U
Yoshizawa 2021. (30)	L	H	H	L	L	L
Tosaki 2017. (31)	L	U	U	L	U	U
Inagaki 2015. (32)	L	L	L	L	U	U
Goldenberg 2017. (33)	L	L	L	L	U	U
Gantz 2017. (34)	L	L	L	L	L	L

L: low risk of bias; U: unclear of bias; H: high risk of bias.

### Result of meta-analysis

#### Primary evaluation indicators

**Figure 2.**
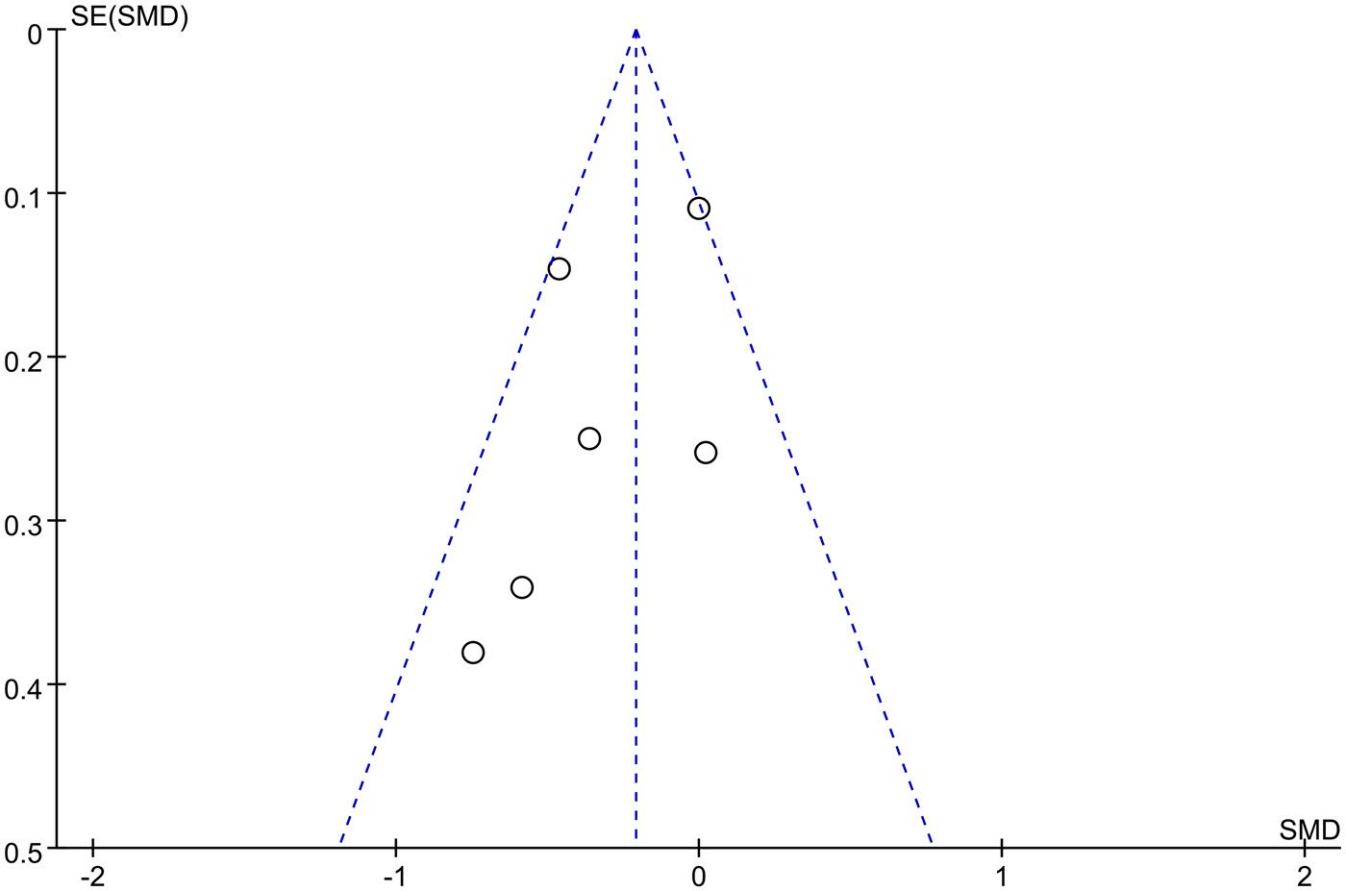
Funnel-plot for assessment of publication bias in HbA1c (%).

During follow-up, the primary evaluation indicators included medication adherence, treatment satisfaction score (DTSQ total score), changes from baseline levels in HbA1c percentage, GA, and fasting plasma glucose among patients switching to once-weekly and those continuing once-daily DPP-4 inhibitors. Among the five indicators, HbA1c percentage was reported in six RCTs [28–32,34], enrolling 715 patients. The heterogeneity test was moderate (*I*^2^ = 68%); accordingly, REM analysis was used, revealing a statistically significant difference between the two groups (MD = −0.21, 95% CI: −0.42 to −0.01, *p* < 0.05). However, the scattered distribution of the literature included in the funnel plot is uneven, and the symmetry is poor, potentially posing a risk of publication deviation (Figure 2), but no statistically significant difference was observed in other indicators (Table 3) (details were shown in Supplementary Material 2).

**Table 3.** Meta-analysis results of change from baseline to end of study in primary indicators.

Outcome index	Studies	Participants	Q test		Statistical model	OR or MD	95%CI	*P-*value
			I^2^	p-value				
HbA1c (%)*	6^[^^28–32^^,^^34^^]^	715	68	0.007	REM	−0.21	−0.42, (−0.01)	0.04
GA	3^[^^30–32^^]^	289	78	0.01	REM	−1.83	−4.46 0.80	0.17
FPG	4^[^^28^^,^^32–34^^]^	1201	87	<0.0001	REM	−0.33	−0.84, 0.18	0.21
DTSQ total score (excluding Q2 and Q3)	4^[^^27–30^^]^	418	0	0.92	FEM	0.70	−0.63, 2.03	0.30
Medication adherence	2^[^^27^^,^^29^^]^	338	9	0.29	FEM	1.27	0.33, 4.81	0.73

* <0.05; HbA1c: glycosylated hemoglobin A1c; GA: glycoalbumin; FPG: fasting blood glucose; DTSQ: diabetes treatment satisfaction questionnaire. The DTSQ scores were analyzed separately for treatment satisfaction and perceived hyperglycemia/hypoglycemia. Treatment satisfaction was estimated by scoring questions 1 (Satisfied), 4 (Convenient), 5 (Flexible), 6 (Understanding), 7 (Recommend) and 8 (Continue) of the DTSQ. Perceived hyperglycemia and hypoglycemia were expressed as the scores of questions 2 and 3, respectively; OR: odds ratio; MD: mean difference.

#### Secondary evaluation indicator

**Figure 3.**
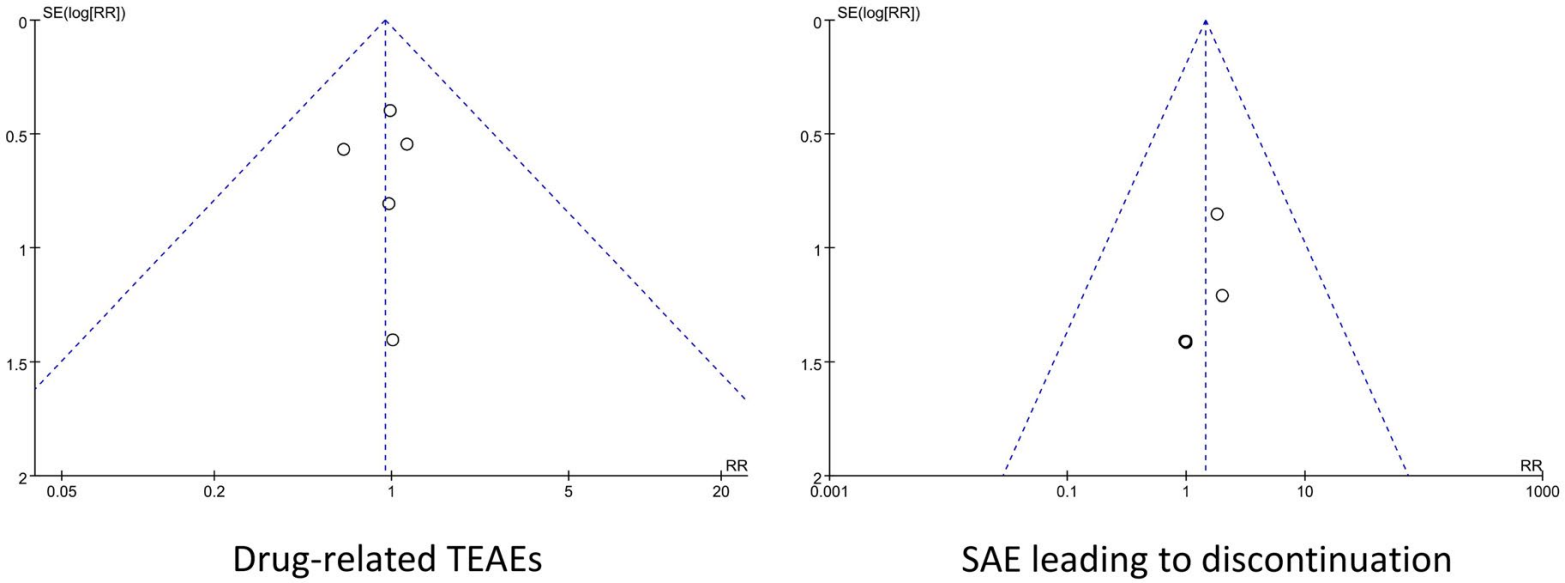
The publication bias evaluation funnel-plot of the incidence of drug-related TEAEs and SAE leading to discontinuation in two groups and its.

The incidence of adverse drug reactions > 2% during the weekly versus daily DPP-4 inhibitor control treatment for T2DM (including the post-unblinding follow-up and expansion period) and the incidence of trial discontinuation owing to SAE were summarized and evaluated. The safety indicators meeting the predefined statistical criteria comprised the analysis of the total incidence of drug-related TEAEs and the incidence rates of infections and infestations, gastrointestinal disorders, musculoskeletal and connective tissue disorders, and viral upper respiratory tract infections (Table 4). Among them, drug-related TEAEs and SAEs, leading to discontinuation of the included articles, were symmetric and well-distributed, suggesting a low risk of publication bias (Figure 3) (details were shown in Supplementary Material 3).

**Table 4.** The secondary indicators were combined with the results of meta-analysis.

	Incidence rate (%)		Studies	Q test		Statistical model	RR	95%CI	P-value
	Once-weekly DPP-4	Daily DPP-4		I^2^	p-value				
Drug-related TEAEs	3.69	3.90	5^[^^27^^,^^29^^,^^32–34^^]^	0	0.96	FEM	0.94	0.57, 1.56	0.81
SAE leading to discontinuation	1.19	0.67	5^[^^27^^,^^29^^,^^32–34^^]^	0	0.98	FEM	1.44	0.52, 4.03	0.48
Infections and infestations	11.16	9.06	4^[^^27^^,^^32–34^^]^	69	0.02	REM	1.12	0.59, 2.10	0.73
Gastrointestinal disorders	2.15	2.78	4^[^^27^^,^^32–34^^]^	0	0.68	FEM	0.77	0.39, 1.49	0.43
Musculoskeletal and connective tissue disorders*	4.13	0.96	3^[^^27^^,^^32^^,^^33^^]^	0	0.55	FEM	2.63	1.18, 5.83	0.02
Viral upper respiratory tract infection	3.51	3.55	3^[^^27^^,^^33^^,^^34^^]^	0	0.47	FEM	0.99	0.55, 1.79	0.97

* <0.05; TEAE: treatment-emergent adverse event; SAE: serious adverse event; RR: risk ratio; FEM: fixed effects model; REM: random effects model.

#### Sensitivity analysis

In REM, sensitivity analyses were conducted for HbA1c (%), GA, FPG, and infection and infestation indicators. For each indicator, the studies with the highest and lowest weights were excluded. The P-value of the HbA1c (%) indicator changed before and after exclusion, suggesting unstable results, while the P-values of GA, FPG, and infection and infestation remained unchanged before and after exclusion, suggesting stable results. However, limitations in study language, incomplete grey-literature data, and small sample sizes warrant cautious interpretation (Table 5).

**Table 5.** Sensitivity analysis result.

Effect index	Remove the highest weight				Remove the lowest weight				Check result
	Statistical model	RR or MD	95% CI	p-value	Statistical model	RR or MD	95% CI	p-value	
HbA1c (%)	FEM	−0.28	−0.42, (−0.14)	<0.0001	REM	−0.17	−0.36, 0.02	0.08	instability
GA	REM	−1.81	−7.49, 3.87	0.53	REM	−0.74	−3.75, 2.28	0.63	stabilization
FPG	REM	−0.53	−1.18, 0.12	0.11	REM	−0.29	−0.87, 0.29	0.33	stabilization
Infections and infestations	REM	0.90	0.36, 2.26	0.82	REM	0.98	0.45, 2.14	0.97	stabilization

GA: glycoalbumin; FPG: fasting blood glucose; FEM: fixed effects model; REM: random effects model; MD: mean difference.

#### Descriptive analysis

One ONWARD DPP-4 Study [35] was included in the descriptive analysis of the treatment burden of patients in both groups. This multicenter RCT screened 367 potential participants, of whom 151 were excluded. Among the 151 excluded participants, 115 did not meet the eligibility criteria, 25 did not participate, and 11 did not register because the planned number of participants had already been registered before the registration. Finally, 216 participants were registered and randomly grouped. The primary endpoint was the change in the Diabetes Treatment Burden Questionnaire (DTBQ) score from baseline to week 12. The results showed that the DTBQ subscale, executive ability, and flexibility burden scores significantly decreased in the omarigliptin switch group, with no between-group differences. However, no data on trelagliptin treatment burden have been identified, precluding definitive conclusions of the study.

## Discussion

Initially, treatment satisfaction was the sole criterion in evaluating medication preferences. However, the systematic review revealed a relatively high risk of subjective bias and insufficient rigor in intervention control. Therefore, evaluating patient treatment preferences requires a multidimensional and systematic approach. Based on principles of implementation science and evidence-based medicine [36], a comprehensive evaluation and analysis framework was developed, incorporating medication preference, treatment satisfaction, glycemic control rate (HbA1c percentage, GA, FPG), treatment burden, and the incidence of drug-related adverse events as effect indicators. Among them, quantitative meta-analysis was used for medication preference, treatment satisfaction, glycemic control rate, and drug-related adverse event incidence, while descriptive analysis was used to assess treatment burden. This study represents the first systematic review and multidimensional evaluation of once-weekly versus once-daily DPP-4 inhibitor preferences for patients with T2DM. The initial literature search encompassed RCTs, cross-sectional surveys, and prospective and retrospective cohort studies. Through literature retrieval, screening, quality evaluation, heterogeneity analysis, and expanded grey-literature sources, nine studies were identified from 615 papers for systematic analysis. However, heterogeneity assessment revealed that retrospective cohort studies exhibited significant influence on meta-analysis results and poor NOS scores. Owing to low data credibility, these studies were excluded. Consequently, eight RCTs [27–34] were included, Methodological quality assessment of the included literature revealed that four studies [27–29,31] had a high risk of bias in participant and personnel blinding. Furthermore, the studies by Ishii [27] and Yoshizawa [31] also exhibited a high risk of bias in allocation concealment. No high risk of bias was identified in selective reporting or other domains. And all of this showed that some limitations existed in the methodological quality.

Although the overall quality-of-life scores did not change significantly among elderly patients with polypharmacy, subgroup analysis [37] suggests significant treatment satisfaction in those administered ≤ 2 drug types or age < 65 years. Overall, the introduction of once-weekly DPP-4 inhibitors has expanded treatment options for T2DM, aligning with the expectations of patients with diabetic to ‘reduce the frequency of medication.’ Adhering to PRISMA (2020) guidelines, this systematic review provides relatively comprehensive evidence showing no significant differences in medication adherence and treatment satisfaction between both groups (*p* > 0.05). These findings indicate that patients with T2DM accustomed to using once-daily DPP-4 inhibitors exhibit no marked preference for once-weekly dosing. Behavioral psychology suggests that, when no significant clinical benefit is observed, patients typically avoid changing treatment regimens or using unfamiliar drugs [38]. The present analysis—showing no significant differences in medication adherence and treatment satisfaction between weekly-switch and daily-continue DPP-4 inhibitor groups—aligns with this cognitive-behavioral characteristic in patients with chronic diseases. However, in a meta-analysis based on 6 RCTs, patients who switched to once-weekly DPP-4 inhibitor therapy had a significantly lower percentage of HbA1c (MD = −0.21, 95% CI [−0.42, −0.01]) compared to the baseline level, and this difference is worthy of further attention. Despite sensitivity analyses and consideration of publication bias, HbA1c percentage remains an important indicator of therapeutic benefit in diabetes management. DPP-4 inhibitors administered once-weekly demonstrated a hypoglycemic effect consistent with early Phase III data, reducing HbA1c percentage by 0.5 − 1.0% as monotherapy or in combination [31]. Noninferiority comparisons against once-daily DPP-4 inhibitors over 24–52 weeks yielded HbA1c percentage reductions of approximately −0.6% to −0.8%, with comparable decreases in fasting and postprandial blood glucose [39–41], which was highly consistent with our research results.

T2DM exhibits the characteristic of ‘dual-track progression and mutual deterioration’ on cardiovascular and hepatic systems. Patients with T2DM face a 2 to 4-fold higher risk of coronary heart disease, myocardial infarction, stroke, and heart failure than non-diabetic patients, and approximately 70% of T2DM-related deaths are attributed to cardiovascular disease [42]. In addition, hepatic dysregulation of glucose metabolism, nonalcoholic fatty liver disease, and insulin resistance exhibit bidirectional causality [43]. ‘Glucose-liver co-treatment’ [44,45] have progressed from the conceptual stage to clinical application. Therefore, T2DM treatment extends beyond glycemic control and pancreatic β-cell function improvement to encompass comprehensive modulation of blood glucose, blood pressure, blood lipids, and weight, aiming to simultaneously reduce the risk of cardiovascular events and liver decompensation. This review did not yield valid statistical data on liver injury and adverse cardiovascular events (only adverse reactions with an incidence > 2% were included in the statistical analysis). Among eight RCTs comprising 1,575 patients, only four clearly defined adverse events statistically; for example, ‘gastrointestinal disorders,’ ‘musculoskeletal and connective tissue disorders,’ ‘viral upper respiratory tract infections,’ ‘infections and infestations,’ while ‘drug-related TEAEs’ and ‘SAE leading to discontinuation’ were not reported in the original trials. Therefore, it remains inconclusive whether once- weekly or once-daily administration of a DPP-4 inhibitor confers greater benefit to hepatic and cardiovascular outcomes. However, the current analysis indicates that, except for the incidence of ‘musculoskeletal and connective tissue disorders,’ which showed a statistically significant difference between groups, no significant differences were observed between both groups across the five analyzed indicators. These findings suggest that once-weekly DPP-4 inhibitors may be associated with a relatively higher risk of ‘musculoskeletal and connective tissue disorders’, while the risk of other adverse events appears comparable between the two groups. Therefore, in the analysis of safety evaluation indicators, no significant intergroup differences were observed regarding patient treatment preferences. Overall, once-weekly and once-daily DPP-4 inhibitors demonstrate comparable efficacy in glycemic control, safety profiles, medication adherence, and treatment satisfaction, as well as perceived treatment burden in T2DM management. Patients exhibited no absolute treatment preference between once-weekly or once-daily DPP-4 inhibitors, with choices primarily influenced by individual needs. Conversely, factors influencing treatment preference may be predominantly clinical and rest with the healthcare provider, who must make comprehensive judgments and considerations based on the specific condition of the individual patient.

## Limitations

Evaluation indicators were developed based on RCT, incorporating medication adherence, treatment satisfaction, glycemic control level, and adverse drug reaction incidence as core metrics. The methodology adhered to PRISMA (2020) guidelines, ensuring rigor and transparency. However, this study has some limitations. First, significant heterogeneity existed in the quality and quantity of evaluation indicators across included studies. This variability may affect the consistency of the outcomes. Second, the original data predominantly consist of statistically significant positive findings, while non-significant or negative outcomes were not reported. This may have introduced a ceiling effect and limited the robustness of the sensitivity analysis. Third, variation in the units of measurement across studies may have introduced statistical biases during the data conversion process, such as rounding and retaining values to three decimal places. In addition, some included literature adopted non-blinded designs, and approximately 50% received low scores for blinding and allocation concealment, raising concerns regarding internal validity and integrity of the original data. Fourth, cultural/device context and external validity: A large share of included trials was conducted in Japan, which undermines external validity. Regional variations in healthcare systems, patient demographics (e.g. age, genetics), treatment practices (e.g. dosage modification, concurrent medications), and regulatory standards for medical products are substantial. For example, Japanese patients differ from Western or other Asian populations in dietary patterns, healthcare accessibility, and baseline disease severity—factors that may drive heterogeneous treatment responses. Consequently, study findings may not be directly generalized to non-Japanese cohorts, restricting the global applicability of our conclusions. Fifth, limited power for rare AEs and adherence endpoints: The pooled sample size lacked sufficient statistical power to detect rare AEs (incidence <5%) and evaluate treatment adherence endpoints. This increases type II error risk for rare safety events (potentially underestimating hazards) and reduces reliability of conclusions on adherence-a key driver of chronic disease treatment efficacy. In addition, for indicators analyzed using the REM, the sensitivity analysis indicates that excluding studies with significantly different weight ratios did not significantly change the direction of the pooled effect, suggesting that our overall results are robust. A further limitation of the existing research is the lack of sufficient, high-quality, long-term clinical follow-up data (exceeding three years), which may contribute to a ceiling effect and potentially obscure the true therapeutic preference results. Therefore, clear recommendations cannot be made until larger-scale, high-quality longitudinal studies are conducted.

## Conclusion

This study represents the systematic review to comprehensively assess treatment preferences for once-weekly and once-daily DPP-4 inhibitors in T2DM, using multidimensional effect indicators—medication compliance, treatment satisfaction, glycemic control levels, and the incidence of adverse drug reactions. Compared with patients who continued using once-daily DPP-4 inhibitors, those who switched to once-weekly regimens exhibited improvements in HbA1c percentage levels and a higher score of treatment satisfaction. Given the study limitations—including reliance on short-term RCTs, a small pooled sample size, substantial heterogeneity, and inconsistent study designs—further research is warranted to validate the observed improvements in HbA1c levels. However, this benefit was accompanied by an increased risk of musculoskeletal and connective tissue disorders. Regarding treatment burden, medication adherence, and therapeutic effect, no differences were observed between once-weekly or once-daily regimens, which appears to be influenced primarily by individual patient preference and the clinical judgement of physicians.

## Supplementary Material

Supplemental Material

Supplementary_materials.zip

## Data Availability

All authors confirm that all data used in this study were obtained from published randomized controlled trial articlesin public databases, and all data and results are presented in the main text and supplementary files. Reasonable requests for the original statistical analysis data can be addressed to the corresponding author.
