# Supplementary material for: Treatment preference for once-weekly versus once-daily DPP-4 inhibitors in patients with type 2 diabetes mellitus: a systematic review and meta-analysis of randomized controlled trials
Source: Ann Med. 2025 Dec 26;58(1):2603036. doi: 10.1080/07853890.2025.2603036 (PMC12777819; doi:10.1080/07853890.2025.2603036)
Supplement: Supplemental Material [file IANN_A_2603036_SM3916.docx]

**Supplementary materials 1.** Risk of bias graph and summary.

**Supplementary materials 2.** Meta-analysis forest plot of the five primary outcome.

**Supplementary materials 3.** Meta-analysis forest plot of the six secondary outcome.
