# Supplementary figures and images for: Treatment preference for once-weekly versus once-daily DPP-4 inhibitors in patients with type 2 diabetes mellitus: a systematic review and meta-analysis of randomized controlled trials
Source: Ann Med. 2025 Dec 26;58(1):2603036. doi: 10.1080/07853890.2025.2603036 (PMC12777819; doi:10.1080/07853890.2025.2603036)

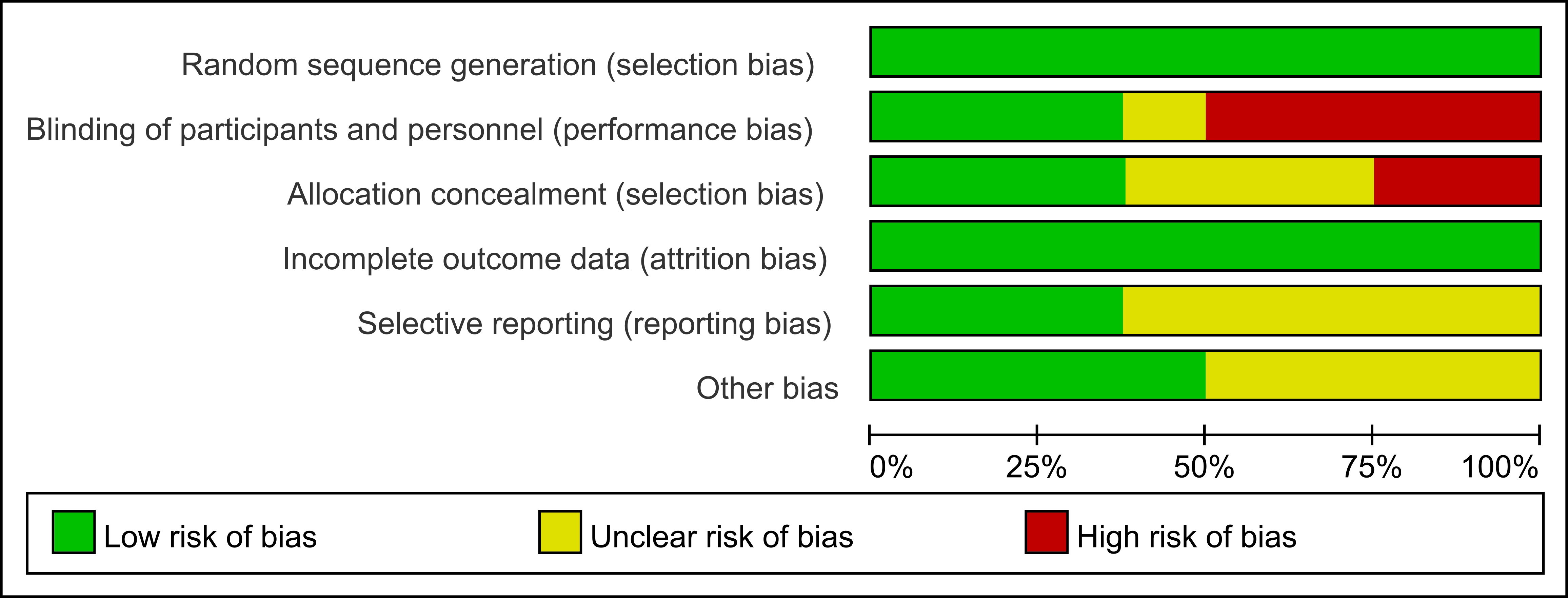

Supplement: Supplementary_materials.zip [file IANN_A_2603036_SM4326.zip › Supplementary materials/Supplementary materials 1_Risk of bias graph.tif]

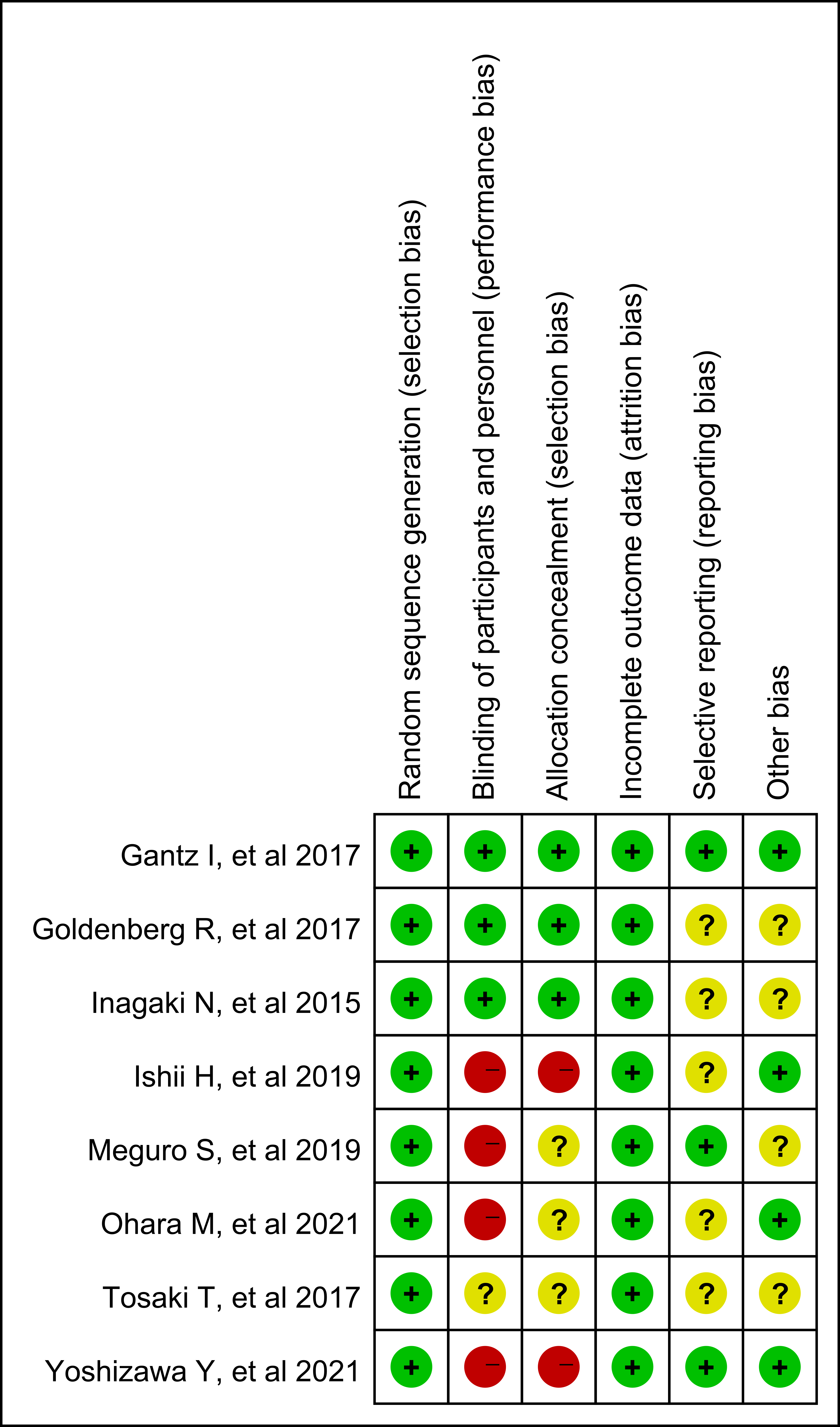

Supplement: Supplementary_materials.zip [file IANN_A_2603036_SM4326.zip › Supplementary materials/Supplementary materials 1_Risk of bias summary.tif]

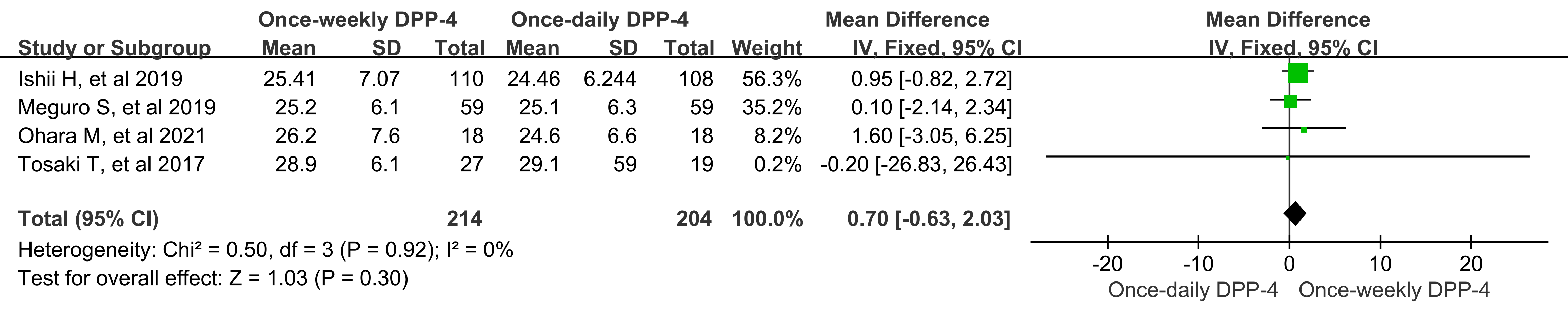

Supplement: Supplementary_materials.zip [file IANN_A_2603036_SM4326.zip › Supplementary materials/Supplementary materials 2_DTSQ total score.tif]

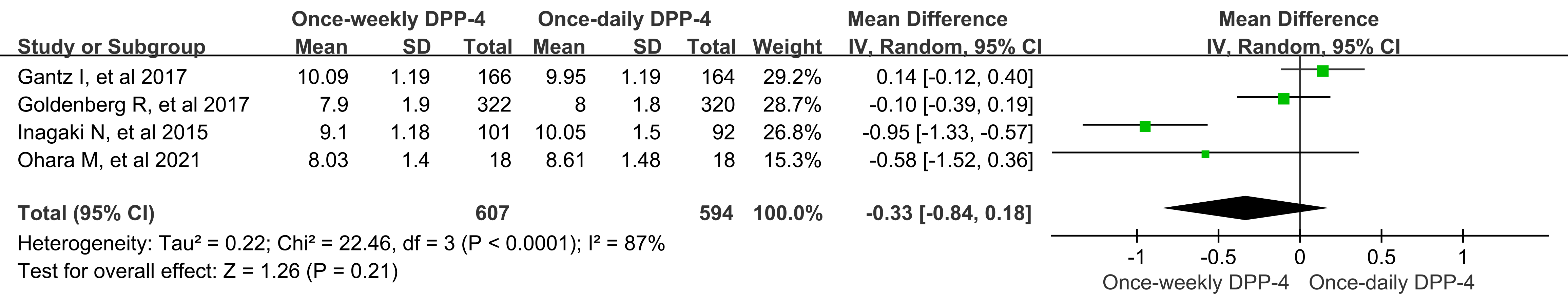

Supplement: Supplementary_materials.zip [file IANN_A_2603036_SM4326.zip › Supplementary materials/Supplementary materials 2_FPG.tif]

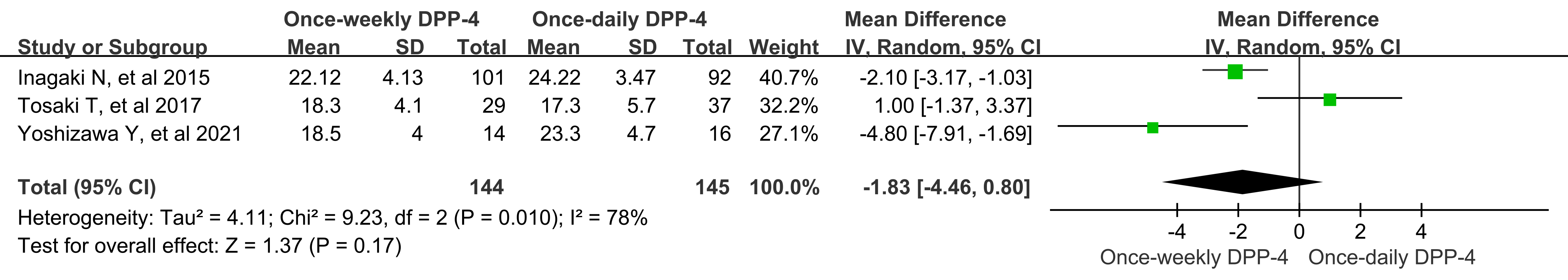

Supplement: Supplementary_materials.zip [file IANN_A_2603036_SM4326.zip › Supplementary materials/Supplementary materials 2_GA.tif]

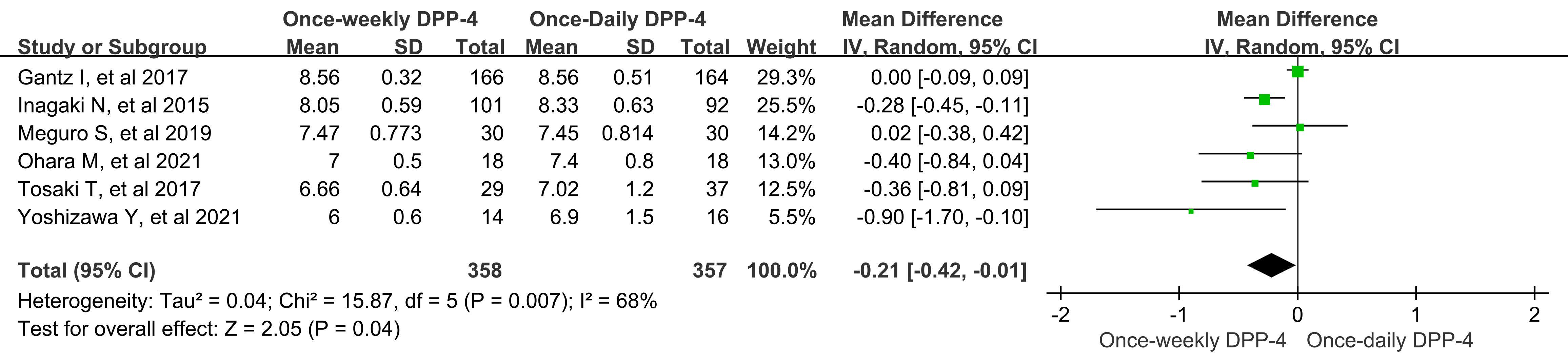

Supplement: Supplementary_materials.zip [file IANN_A_2603036_SM4326.zip › Supplementary materials/Supplementary materials 2_HbA1c percentage.tif]

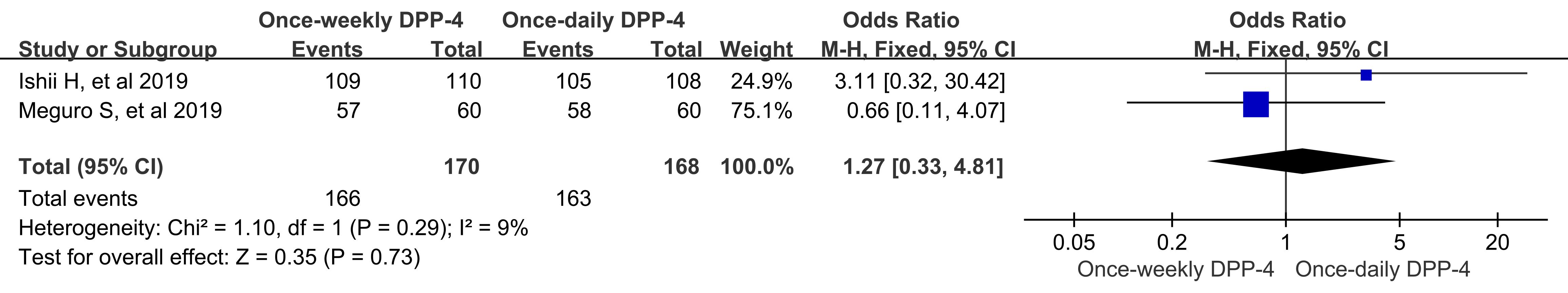

Supplement: Supplementary_materials.zip [file IANN_A_2603036_SM4326.zip › Supplementary materials/Supplementary materials 2_Medication adereence.tif]

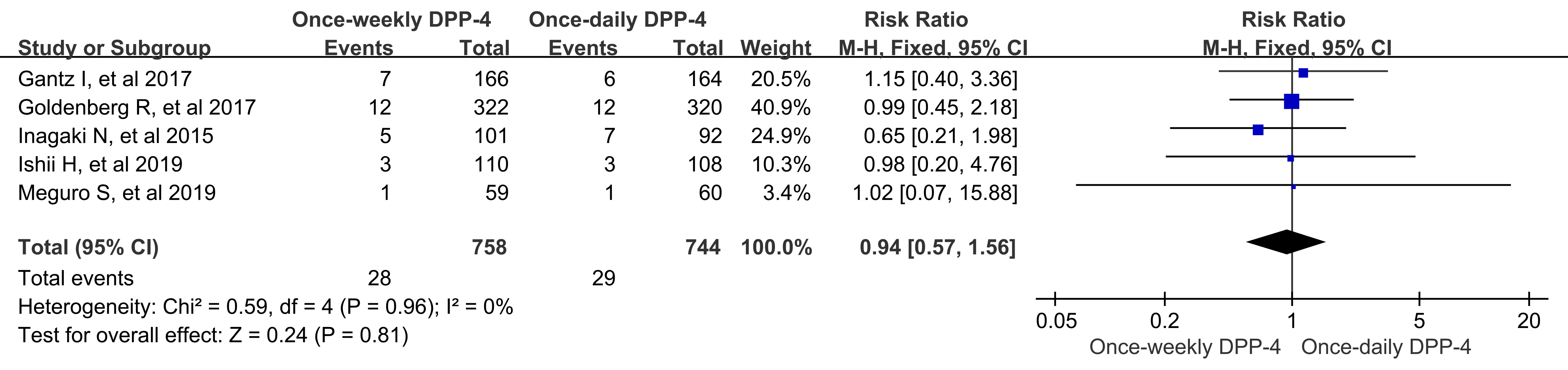

Supplement: Supplementary_materials.zip [file IANN_A_2603036_SM4326.zip › Supplementary materials/Supplementary materials 3_Drug related TEAEs.tif]

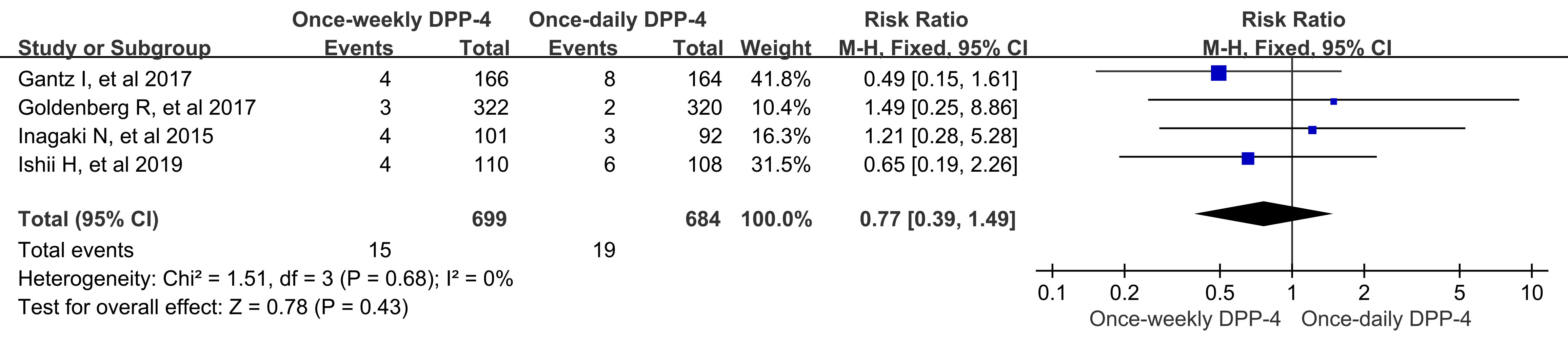

Supplement: Supplementary_materials.zip [file IANN_A_2603036_SM4326.zip › Supplementary materials/Supplementary materials 3_Gastrointestinal disorders.tif]

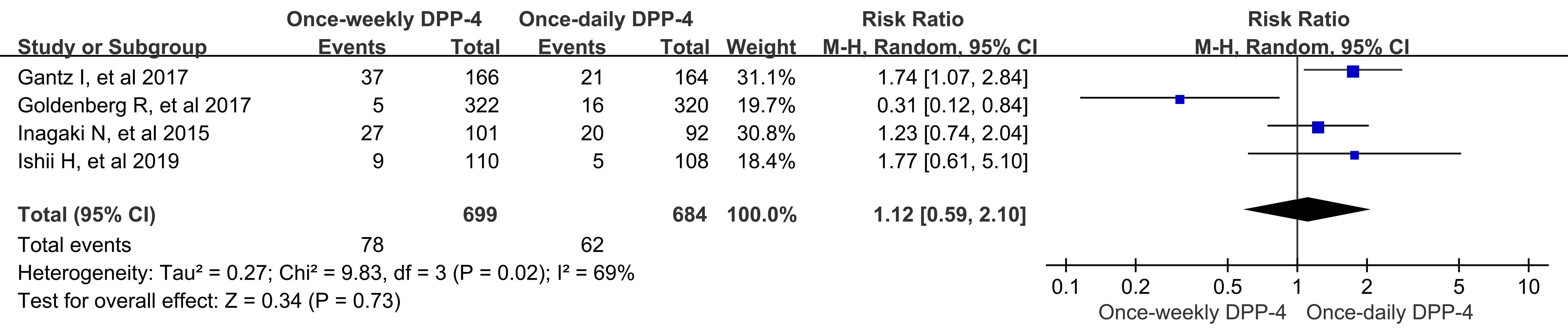

Supplement: Supplementary_materials.zip [file IANN_A_2603036_SM4326.zip › Supplementary materials/Supplementary materials 3_Infections and infestations.tif]

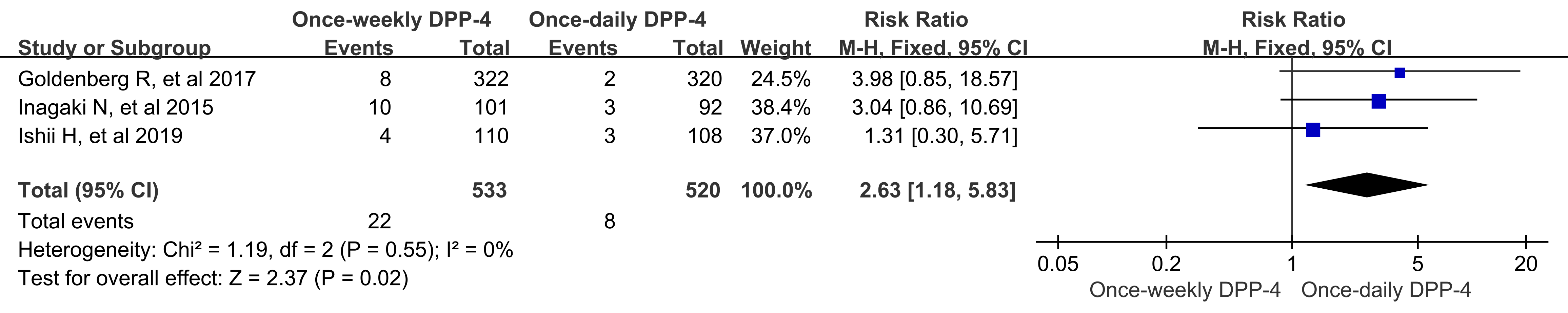

Supplement: Supplementary_materials.zip [file IANN_A_2603036_SM4326.zip › Supplementary materials/Supplementary materials 3_Musculoskeletal and connective tissue disorders.tif]

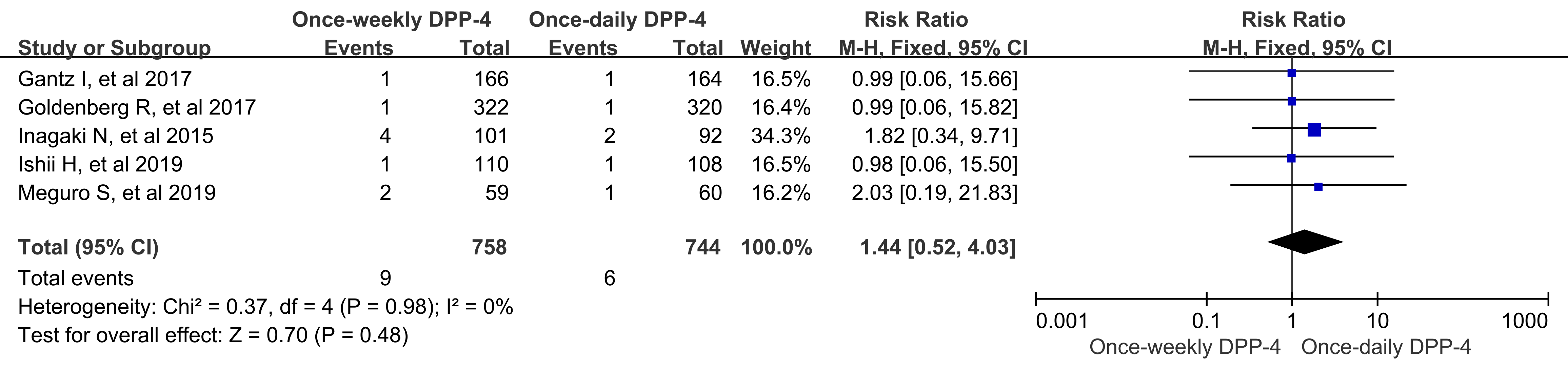

Supplement: Supplementary_materials.zip [file IANN_A_2603036_SM4326.zip › Supplementary materials/Supplementary materials 3_SAE leading to discontinuation.tif]

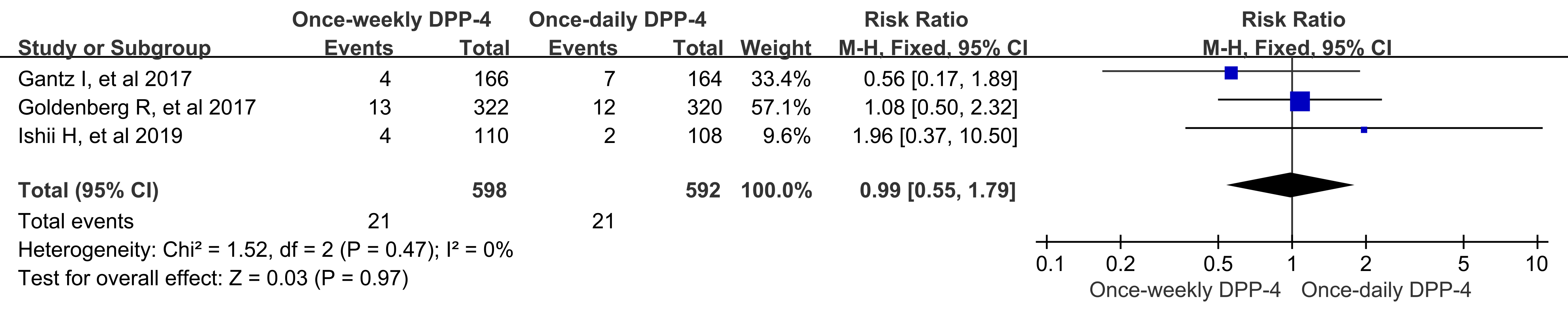

Supplement: Supplementary_materials.zip [file IANN_A_2603036_SM4326.zip › Supplementary materials/Supplementary materials 3_Viral upper respiratory tract infection.tif]
